# Clinical observation of different dosages of dexmedetomidine combined with a target-controlled infusion of propofol in hysteroscopic submucosal myomectomy

**DOI:** 10.3389/fsurg.2022.1025592

**Published:** 2023-01-06

**Authors:** Haibing Li, Qingsong Zhao, Yibing Yu, Wei Li

**Affiliations:** Department of Anesthesiology, Shanghai Key Laboratory of Maternal Fetal Medicine, Shanghai Institute of Maternal-Fetal Medicine and Gynecologic Oncology, Shanghai First Maternity and Infant Hospital, School of Medicine, Tongji University, Shanghai, China

**Keywords:** dexmedetomidine, propofol, hysteroscopy, anesthesia, hysteroscopic surgery

## Abstract

**Objective:**

This study aimed to explore the clinical effects of different dosages of dexmedetomidine (Dex) combined with a target-controlled infusion of propofol in hysteroscopic submucosal myomectomy.

**Methods:**

Ninety patients who underwent hysteroscopic submucosal myomectomy between September 2021 and March 2022 were enrolled and randomly divided into three groups, with 30 patients in each group. Patients in Groups A, B, and C received injections of 0.25, 0.5, or 0.75 µg/kg of Dex, respectively, by intravenous pump over 10 min. After this time, a maintenance dosage of 0.5 µg/kg/h was administered by intravenous infusion until the end of the surgery. Anesthesia was induced using 1.5 mg/kg of propofol and 0.3 µg/kg of sufentanil that were introduced through a laryngeal mask. The plasma concentration of propofol was maintained at 3 µg/ml by target-controlled infusion until the end of the surgery. The mean arterial pressure (MAP), heart rate (HR), and electroencephalographic bispectral index (BIS) were observed when the patient entered the operating room (T0), after catheter indwelling for anesthesia (T1), at the time of cervical dilation (T2), at the time of hysteroscopic surgery (T3), and at the end of the surgery (T4) in all three groups. The total dosage of propofol for induction and maintenance, anesthesia awakening time, orientation recovery time, Visual Analog Scale (VAS) score of the post-awakening uterine contraction pain, and adverse reactions were recorded.

**Results:**

The intraoperative reductions of MAP and HR in patients were significant in Group C when compared with those in Groups A and B (*P *< 0.05), and BIS was significantly lower in Group C at T2 and T3 when compared with the baseline measurement at T0 (*P *< 0.05). The dosage of propofol was significantly higher for Group A than for Groups B and C (*P *< 0.05). The anesthesia awakening time and orientation recovery time were significantly longer for patients in Group C when compared with patients in Groups A and B (*P *< 0.05). Within 5–30 min after awakening, the VAS scores in Groups B and C were significantly lower than those for Group A (*P *< 0.05). The incidence of adverse reactions in Group B was significantly less than that for Groups A and C (*P *< 0.05).

**Conclusion:**

The continuous pumping of 0.5 µg/kg of Dex combined with a target-controlled infusion of propofol in hysteroscopic submucosal myomectomy resulted in positive anesthetic and analgesia effects and fewer adverse reactions. It therefore has high clinical significance.

## Introduction

The advantages of hysteroscopic submucosal myomectomy include less surgical trauma and faster postoperative recovery (1). Mechanical operations like intraoperative cervical dilatation, uterine traction, and negative pressure suction may cause abdominal pain, nausea, and irritability, which may result in discomfort to patients and may even be life threatening (2). The anesthesia protocol choice is closely tied to the safety and comfort of patients and the prognosis of hysteroscopic surgery (3). Propofol is widely applied in outpatient examination and treatment because it takes effect quickly, has a short action time, and results in a high level of patient consciousness. As an opioid receptor agonist, sufentanil is a fentanyl derivative with the strongest analgesic effect. It has a long action time and little impact on the cardiovascular system and hemodynamics. Dexmedetomidine (Dex) is a highly selective α2-adrenergic agonist that activates the locus coeruleus receptor of the brainstem, inhibits sympathetic activity, and produces sedative and analgesic effects that have little impact on the respiratory and circulatory systems. It can reduce the dosage of drugs required in combination, thereby diminishing the side effects of anesthesia (4). In the present study, the appropriate dosage of Dex for hysteroscopic submucosal myomectomy was investigated by observing the intraoperative anesthetic effects, hemodynamic changes, and adverse reactions of different dosages of Dex when used in combination with a target-controlled infusion of propofol.

## Materials and methods

### Subjects

A total of 90 patients who underwent hysteroscopic submucosal myomectomy between September 2021 and March 2022 were enrolled in the study. These patients were classified as grades I–III in the American Society of Anesthesiologists (ASA) system, aged 20–40 years, and had body weights within the range of 50–70 kg. None of the patients showed abnormalities in the preoperative laboratory examinations. The patients were randomly divided into three groups (Groups A–C) using the random number table method; each group contained 30 patients.

### Size and classification of submucous hysteromyoma

According to the maximum diameter of the myoma measured by B-ultrasound during the operation, in 21 of the 90 patients the maximum diameter was <2 cm, in 52 cases it was 2–4 cm, and in 17 cases it was >4 cm. Based on the nine-type classification method of hysteromyoma put forth by the International Federation of Gynecology and Obstetrics, submucous hysteromyomas are divided into three types according to the relationship between the hysteromyoma and the myometrium. Type 0 is a pedunculated submucous myoma, type 1 is a sessile submucous myoma (the tumor body expands to the muscle layer ≤50%), and type 2 is a sessile submucous myoma (the tumor body expands to the muscle layer >50%). Of the 90 patients in this clinical study, 16 had submucous hysteromyomas that were type 0, 61 had type 1, and 13 had type 2.

### Surgical instruments and materials

The surgical equipment was TV hysteroscope equipment purchased from WOLF company in Germany. The distending medium was a 0.9% normal saline solution, and the flow was 120 ml/min. The distending pressure was 90–110 mmHg (1 mmHg = 0.133 kPa). The output power of the bipolar electrode was 40–60 W for electrocoagulation and 80–100 W for cutting. A Toshiba ultrasonic diagnostic instrument (SSA-550A) was employed to monitor the patients during the operation.

### Anesthetic methods

Prior to surgery, the patients went through routine fasting. Venous access to the upper limb was established after entering the operating room. The lactated Ringer's solution was first administered during the induction of anesthesia, then at 6–8 ml/(kg·h) until the end of surgery. The blood pressure (BP), heart rate (HR), pulse oxygen saturation (SpO_2_), and electroencephalographic bispectral index (BIS) were routinely monitored.

Patients in Groups A–C received injections of 0.25, 0.5, or 0.75 µg/kg of Dex, respectively, by intravenous pump as a loading dose over 10 min. After this time, a maintenance dosage (0.5 µg/kg/h) of Dex was administered by intravenous infusion until the end of the surgery. Anesthesia was induced using 1.5 mg/kg of propofol and 0.3 µg/kg of sufentanil introduced through a laryngeal mask. The plasma target concentration of propofol was maintained at 3 µg/ml by target-controlled infusion until the end of the surgery. After surgery, patients were sent to the resuscitation room for monitoring and observation.

### Monitoring parameters

In all three groups, the BP, HR, SpO_2_, and BIS of the patients were observed when they entered the operating room (T0), after the catheter indwelling for anesthesia (T1), at the time of cervical dilation (T2), at the time of hysteroscopic surgery (T3), and at the end of the surgery (T4). The total dosage of propofol for induction and maintenance, anesthesia awakening time, orientation recovery time, Visual Analog Scale (VAS) score of post-awakening uterine contraction pain, and adverse effects were recorded.

### Statistical analysis

The SPSS™ Statistics v22.0 software was adopted for statistical analysis. The measurement data were expressed as the mean ± standard deviation ($\bar{x}\pm s$). The *χ*^2^ test was used for comparison between the three groups, and the *t*-test was adopted for pairwise comparison. The countable data were expressed as the number of cases or percentages. A value of *P *< 0.05 was considered statistically significant.

## Results

### Pain management

Technological advances have led to smaller hysteroscopes and ancillary instrumentation, which has made it possible to carry out procedures without the need for anesthesia—or with only a local genital tract anesthesia. The feasibility of conducting procedures without general or regional anesthesia is dependent upon several clinical and non-clinical factors. These include the type of procedure, patient preferences, clinician expertise, the available instrumentation and infrastructure, and how health services are reimbursed and regulated.

Technological advances have led to the miniaturisation of hysteroscopes and ancillary instrumentation, which has facilitated the conduct of procedures without the need for anaesthesia or with the use of local genital tract anaesthesia alone. The feasibility of conducting procedures without the need for conventional general or regional anaesthesia is dependent upon several factors both clinical and non-clinical and these include the type of procedure, patient preferences, clinician expertise, the available instrumentation and infrastructure and how health services are reimbursed and regulated (5, 6).

Thus, the management of pain is a key consideration when undertaking hysteroscopic procedures and needs to be clearly and consistently reported. A hierarchical description of pain management, consisting of five levels, is recommended (see Table 1).

**Table 1:** Levels of pain management used during hysteroscopic procedures^a^.

Level 1	No medication or the use of oral nonsedative medication
Level 2	Local anesthetic to the genital tract
Level 3	Conscious sedation
Level 3 (a)	Oral or inhalational medications with a sedative effect
Level 3 (b)	Parenteral medication with a sedative effect
Level 4	Regional anesthesia
Level 5	General anesthesia

^a^ Pain management should be defined according to the highest level of intervention used to control pain if combined therapies are used.

### Comparison of the general characteristics between the groups

When comparing the three groups, the differences in age, body weight, body mass index, ASA grade, and operation duration were not statistically significant (*P *> 0.05; see Table 2).

**Table 2:** Comparison of the general characteristics and operation duration among the three groups of patients.

Group	The number of cases	Age (Year, $\bar{x}\pm s$)	Weight (kg, $\bar{x}\pm s$)	BMI (kg/m^2^, $\bar{x}\pm s$)	Operation duration (min, $\bar{x}\pm s$)
A	30	32.1 ± 6.5	61.3 ± 8.1	20.4 ± 3.4	25.9 ± 5.3
B	30	30.7 ± 9.1	58.9 ± 7.3	19.5 ± 2.5	26.7 ± 5.4
C	30	31.4 ± 7.2	61.3 ± 6.7	20.1 ± 3.2	25.8 ± 4.7
*F*		0.285	1.725	1.352	1.754
*P*		0.083	0.073	0.107	0.141

### Comparison of mean arterial pressure (MAP), heart rate, and electroencephalographic bispectral index between the groups

When comparing the three groups, the differences in baseline MAP, HR, and BIS were not statistically significant (*P *> 0.05). The results of pairwise comparison for Groups A and B revealed that the MAP, HR, and BIS recorded at T1, T2, and T3 were not statistically different when compared with the baseline measurements recorded at T0. However, in Group C, when compared with the measurements taken at T0, the MAP, HR, and BIS recorded at T2, T3, and T4 decreased significantly (*P *< 0.05). At T2 and T3, BIS was significantly lower than at T0 (*P *< 0.05), but there was no significant change in BIS at T4 (*P *> 0.05). See Table 3.

**Table 3:** Comparison of MAP, HR, and BIS at different time points among the three groups of patients.

Group	T0	T1	T2	T3	T4
MAP (mmHg)					
A	82.7 ± 7.2	79.7 ± 7.5	80.1 ± 6.3	77.4 ± 6.1	85.2 ± 4.5
B	82.6 ± 6.3	80.1 ± 6.5	75.4 ± 4.1	72.5 ± 5.4	82.5 ± 4.3
C	81.9 ± 5.7	81.2 ± 5.4	70.1 ± 5.4*	69.5 ± 5.9*	81.4 ± 3.6
*F*	0.249	−0.051	1.193	−0.609	−1.179
*P*	0.803	0.960	0.041	0.016	0.246
HR (Beats/min)					
A	83 ± 14	79 ± 12	80 ± 11	81 ± 9	84 ± 13
B	84 ± 17	80 ± 17	73 ± 9	66 ± 6	83 ± 11
C	85 ± 15	80 ± 13	69 ± 7*	63 ± 8*	80 ± 12
*F*	1.618	0.167	0.386	−0.271	−1.521
*P*	0.114	0.863	0.010	0.000	0.352
BIS					
A	97.3 ± 0.6	49.7 ± 6.4	50.9 ± 3.1	50.7 ± 4.3	89.1 ± 4.5
B	97.7 ± 0.5	48.1 ± 4.5	48.4 ± 4.1	47.7 ± 5.1	88.3 ± 4.0
C	96.9 ± 0.3	46.8 ± 5.2	42.3 ± 4.9*	43.2 ± 4.7*	86.9 ± 5.1
*F*	0.843	1.386	−0.271	−1.337	−1.523
*P*	0.402	0.173	0.031	0.027	0.136

Compared between those at T1, T2, T3, and T4 with those at T0 within the group, *P < 0.05.

### Comparison of the total dosage of propofol for induction and maintenance, awakening time, and orientation recovery time between the groups

The total dosage of propofol in Group A was higher than in Groups B and C, and the differences were statistically significant (*P *< 0.05). However, the difference in total dosage of propofol between patients in Groups B and C was not statistically significant (*P *> 0.05). The awakening and orientation recovery times were longer in Group C when compared with Groups A and B, and the differences were statistically significant (*P *< 0.05). However, the differences between Groups A and B for the awakening and orientation recovery times were not statistically significant (*P *> 0.05). See Table 4.

**Table 4:** Comparison of the total dosage of propofol, awaking time, and orientation recovery time among the three groups of patients.

Group	The number of cases	The total dosage of propofol	The awaking time	The orientation recovery time
A	30	353.2 ± 3.6	11.3 ± 1.3	22.5 ± 3.1
B	30	315.3 ± 2.3*	12.2 ± 2.1	25.2 ± 5.1
C	30	285.1 ± 2.7*	13.1 ± 3. 5*,**	31.4 ± 6.7*,**
*F*		6.705	−1.428	−5.402
*P*		0.027	0.036	0.013

Compared between group B, group C with group A., *P < 0.05; Compared between group C and group B, **P < 0.05.

### Comparison of the post-awakening uterine contraction pain between the groups

In Group A, the VAS scores at 5 and 15 min after awakening were higher than the corresponding scores in Groups B and C, and the differences were statistically significant (*P <* 0.05). For the three groups, the differences in the VAS scores at 30 min after awakening were not statistically significant (*P *> 0.05). See Table 5.

**Table 5:** The VAS scores of uterine contraction pain at 5 min, 15 min, and 30 min after awaking among the three groups of patients.

Group	The number of cases	5 min	15 min	30 min
A	30	5.6 ± 0.7	4.9 ± 0.1	3.2 ± 0.1
B	30	4.5 ± 0.3*	3.7 ± 0.3*	1.3 ± 0.2
C	30	4.4 ± 0. 5*	3.3 ± 0.2*	1.3 ± 0.3
*F*		4.326	3.092	−7.121
*P*		0.015	0.004	0.626

Compared between group B, group C with group A, *P < 0.05.

### Comparison of the adverse reactions between the groups

In Group A, three patients had intraoperative frowning and body movement, and two patients experienced postoperative vomiting and headaches. In Group B, there was one patient each with bradycardia and a postoperative reduction in SpO_2_. In Group C, there were three patients with bradycardia, one with hypotension, and one with a postoperative reduction in SpO_2_. The incidence of frowning and body movement, as well as postoperative vomiting and headache, were higher in Group A than in Groups B and C, and the differences were statistically significant (*P *< 0.05). The number of cases with bradycardia was higher in Group C than in Groups A and B, and the differences were statistically significant (*P *< 0.05).

## Discussion

Unlike ordinary hysteroscopy, hysteroscopic submucosal myomectomy is a relatively complex and lengthy procedure with some commonly encountered issues that can affect the outcome of the surgery. These include excessive uterine flexion, cervical stenosis, a history of cervical surgery, uterine adhesions, improper selection of electric knife and roller, and poor experience of the attending surgeon (7). In addition, the intraoperative process of hysteroscopic surgery is more likely to cause vagal reflexes in patients, which increases the risk involved in anesthetic management (8). Furthermore, hysteroscopic surgery is more painful for patients and involves relatively superficial anesthesia (9). It tends to cause frequent body movements in the patient, which affects the surgical operation and may lead to intraoperative uterine perforation or injury to the bowel or bladder (10).

The advantages of propofol are that it acts quickly, it stabilizes the anesthesia when combined with Dex, it is metabolized quickly by patients, and there is almost no drug accumulation or residue (11). It meets the criterion that a patient should awaken quickly after hysteroscopic treatment and is the most widely adopted intravenous anesthetic in clinical practice (12). Sufentanil is a specific μ-opioid receptor agonist with a strong analgesic effect (13). The effects on myocardial oxygen supply and hemodynamics are small, and patients show a low incidence of postoperative respiratory depression (14). Although the combination of propofol and sufentanil has been effective for short and minor operations, the sedative effect is still insufficient (15, 16). Patients undergoing hysteroscopic surgery may suffer from preoperative anxiety, which can lead to high BP and rapid HR when entering the operation room. This, in turn, can lead to increased intraoperative hemorrhage (17). Meanwhile, the application of high dosages of propofol with opioids has a higher risk of postoperative respiratory depression, prolonged anesthesia awakening time, nausea, vomiting, and other adverse reactions (18).

Dex is a highly selective α2-adrenergic agonist that acts centrally on the locus coeruleus to exert sedative and anxiolytic effects, and its unique conscious sedation effect allows patients to be awakened easily (19, 20). It acts on the spinal cord to exert analgesic effects. It permits the dosage of opioids and general anesthetics to be reduced and has no respiratory depressant effect alone (21). Park et al. (22) reported that Dex maintained the perioperative stability of the cardiovascular system in patients, enhanced anesthetic efficacy, and allowed the dosage of propofol to be reduced. Zhang RC's study showed: The operative duration was shorter and the intraoperative bleeding volume was lower in the hysteroscopy than laparoscopy group. The time to complete healing of the muscle layer was shorter in the hysteroscopy than laparoscopy group. The rate of intraoperative complications was lower in the hysteroscopy than laparoscopy group (23). The results of the present study found that the reductions in intraoperative MAP and HR were significant in the 0.75 µg/kg group than in the 0.5 µg/kg group and 0.25 µg/kg group (*P *< 0.05). The total dosage of propofol was significantly higher for patients in Group A (who received 0.25 µg/kg of Dex) when compared with patients in Groups B and C (*P* < 0.05). The awakening and orientation recovery times were significantly longer for patients in Group C (who received 0.75 µg/kg of Dex) when compared with patients in Groups A and B (*P* < 0.05). The VAS scores for patients in Group B (who received 0.5 µg/kg of Dex) and Group C (who received 0.75 µg/kg of Dex) were significantly lower than those for patients in Group A (who received 0.25 µg/kg of Dex) 5–30 min after awakening (*P* < 0.05). The incidence of adverse reactions for patients in Group B (who received 0.5 µg/kg of Dex) was significantly lower than in other two Groups (*P* < 0.05). The response to the injurious stimulus of the hysteroscopic surgery in patients with the intraoperative administration of 0.5 µg/kg of Dex was less pronounced and the intraoperative vital signs were stable.

## Conclusion

The continuous pumping of 0.5 µg/kg/h of Dex combined with a target-controlled infusion of propofol in hysteroscopic submucosal myomectomy resulted in optimal analgesic and sedative effects and less hemodynamic impact on patients. It allowed the dosage of propofol to be reduced, shortened the awakening time for the patients, improved the safety of the anesthesia, and resulted in fewer adverse reactions. Therefore, this research finding has high clinical significance. However, a larger sample size of data is needed to further evaluate the efficacy and safety of this study.

## Data Availability

The original contributions presented in the study are included in the article/Supplementary Material, further inquiries can be directed to the corresponding author/s.

## References

[1] Di Spiezio Sardo A Zizolfi B Calagna G Giampaolino P Paolella F Bifulco G. Hysteroscopic isthmoplasty: step-by-step technique. J Minim Invasive Gynecol. (2018) 25(2):338–9. 10.1016/j.jmig.2017.09.002 28893656

[2] Spanish Infertility SWOT Group (SISG), Checa MA Bellver J Bosch E Espinós JJ Fabregues F Hysteroscopic septum resection and reproductive medicine: a SWOT analysis. Reprod Biomed Online. (2018) 37(6):709–15. 10.1016/j.rbmo.2018.09.013 30527061

[3] Casadei L Piccolo E Manicuti C Cardinale S Collamarini M Piccione E. Role of vaginal estradiol pretreatment combined with vaginal misoprostol for cervical ripening before operative hysteroscopy in postmenopausal women. Obstet Gynecol Sci. (2016) 59(3):220–6. 10.5468/ogs.2016.59.3.220 27200313 PMC4871939

[4] Grape S Kirkham KR Frauenknecht J Albrecht E. Intra-operative analgesia with remifentanil vs. dexmedetomidine: a systematic review and meta-analysis with trial sequential analysis. Anaesthesia. (2019) 74(6):793–800. 10.1111/anae.14657 30950522

[5] Carugno J Grimbizis G Franchini M Alonso L Bradley L Campo R International consensus statement for recommended terminology describing hysteroscopic procedures. Facts Views Vis Obgyn. (2021) 13(4):287–94. 10.52054/FVVO.13.4.037 34647447 PMC9148713

[6] Carugno J Grimbizis G Franchini M Alonso L Bradley L Campo R International consensus statement for recommended terminology describing hysteroscopic procedures. J Minim Invasive Gynecol. (2022) 29(3):385–91. 10.1016/j.jmig.2021.10.004 34648932

[7] Ludwin A Lindheim SR Booth R Ludwin I. Removal of uterine polyps: clinical management and surgical approach. Climacteric. (2020) 23(4):388–96. 10.1080/13697137.2020.1784870 32648824

[8] Kamath MS Kalampokas EE Kalampokas TE. Use of GnRH analogues pre-operatively for hysteroscopic resection of submucous fibroids: a systematic review and meta-analysis. Eur J Obstet Gynecol Reprod Biol. (2014) 177:11–8. 10.1016/j.ejogrb.2014.03.009 24702901

[9] Munro MG Christianson LA. Complications of hysteroscopic and uterine resectoscopic surgery. Clin Obstet Gynecol. (2015) 58(4):765–97. 10.1097/GRF.0000000000000146 26457853

[10] Raz N Feinmesser L Moore O Haimovich S. Endometrial polyps: diagnosis and treatment options—a review of literature. Minim Invasive Ther Allied Technol. (2021) 30(5):278–87. 10.1080/13645706.2021.1948867 34355659

[11] Seangrung R Pasutharnchat K Injampa S Kumdang S Komonhirun R. Comparison of the hemodynamic response of dexmedetomidine versus additional intravenous lidocaine with propofol during tracheal intubation: a randomized controlled study. BMC Anesthesiol. (2021) 21(1):265. 10.1186/s12871-021-01484-6 34717532 PMC8557037

[12] Akhtar S Heng J Dai F Schonberger RB Burg MM. A retrospective observational study of anesthetic induction dosing practices in female elderly surgical patients: are we overdosing older patients? Drugs Aging. (2016) 33(10):737–46. 10.1007/s40266-016-0394-x 27581549

[13] Weber F Prasser C. Investigating propofol-sufentanil interaction using clinical endpoints and processed electroencephalography: a prospective randomized controlled trial. Minerva Anestesiol. (2019) 85(3):271–8. 10.23736/S0375-9393.18.12326-1 29945431

[14] Yu J Xiang B Song Y Chen H Li Y Liu C. ED50 Of propofol in combination with low-dose sufentanil for intravenous anaesthesia in hysteroscopy. Basic Clin Pharmacol Toxicol. (2019) 125(5):460–5. 10.1111/bcpt.13280 31231918

[15] Al-Husban N Aloweidi A Ababneh O. The impact of spinal anesthesia and use of oxytocin on fluid absorption in patients undergoing operative hysteroscopy: results from a prospective controlled study. Int J Womens Health. (2020) 12:359–67. 10.2147/IJWH.S249619 32440230 PMC7212770

[16] Saugel B Bebert EJ Briesenick L Hoppe P Greiwe G Yang D Mechanisms contributing to hypotension after anesthetic induction with sufentanil, propofol, and rocuronium: a prospective observational study. J Clin Monit Comput. (2022) 36(2):341–7. 10.1007/s10877-021-00653-9 33523352 PMC9122881

[17] De Cassai A Boscolo A Geraldini F Zarantonello F Pettenuzzo T Pasin L Effect of dexmedetomidine on hemodynamic responses to tracheal intubation: a meta-analysis with meta-regression and trial sequential analysis. J Clin Anesth. (2021) 72:110287. 10.1016/j.jclinane.2021.110287 33873003

[18] Karmaniolou I Staikou C Surda P. The role of dexmedetomidine as an additive to intravenous regional anesthesia: a systematic review and meta-analysis. Balkan Med J. (2021) 38(3):156–64. 10.5152/balkanmedj.2021.20076 33593724 PMC8880966

[19] Wu X Hang LH Chen YF Wang H Shao DH Chen Z. Remifentanil requirements for preventing motor response to skin incision in healthy women anesthetized with combinations of propofol and dexmedetomidine titrated to similar Bispectral Index (BIS) values. Ir J Med Sci. (2015) 184(4):805–11. 10.1007/s11845-014-1176-2 25085686

[20] Park HY Kim JY Cho SH Lee D Kwak HJ. The effect of low-dose dexmedetomidine on hemodynamics and anesthetic requirement during bis-spectral index-guided total intravenous anesthesia. J Clin Monit Comput. (2016) 30(4):429–35. 10.1007/s10877-015-9735-2 26162785

[21] Le Guen M Liu N Tounou F Augé M Tuil O Chazot T Dexmedetomidine reduces propofol and remifentanil requirements during bispectral index-guided closed-loop anesthesia: a double-blind, placebo-controlled trial. Anesth Analg. (2014) 118(5):946–55. 10.1213/ANE.0000000000000185 24722260

[22] Park S Choi SL Nahm FS Ryu JH Do SH. Dexmedetomidine-remifentanil vs propofol-remifentanil for monitored anesthesia care during hysteroscopy: randomized, single-blind, controlled trial. Medicine (Baltimore). (2020) 99(43):e22712. 10.1097/MD.0000000000022712 33120766 PMC7581053

[23] Zhang RC Wu W Zou Q Zhao H. Comparison of clinical outcomes and postoperative quality of life after surgical treatment of type II submucous myoma via laparoscopy or hysteroscopy. J Int Med Res. (2019) 47(9):4126–33. 10.1177/0300060519858027 31280641 PMC6753532

